# Molecular findings in maxillofacial bone tumours and its diagnostic value

**DOI:** 10.1007/s00428-019-02726-2

**Published:** 2019-12-14

**Authors:** Arjen H.G. Cleven, Willem H. Schreuder, Eline Groen, Herman M. Kroon, Daniel Baumhoer

**Affiliations:** 1 Department of Pathology, Leiden University Medical Center, PO Box 9600, L1-Q, 2300 RC, Leiden, the Netherlands; 2 Department of Oral and Maxillofacial Surgery/Head and Neck Surgery, Amsterdam University Medical Center/Antoni van Leeuwenhoek Hospital, Amsterdam, the Netherlands; 3 Department of Radiology, Leiden University Medical Center, Leiden, the Netherlands; 4 Bone Tumour Reference Centre, Institute of Pathology, University Hospital Basel, University of Basel, Basel, Switzerland

**Keywords:** Maxillofacial bone tumours, Multidisciplinary diagnostic approach, Fibro-osseous lesions, Genetic aberrations

## Abstract

According to the WHO, mesenchymal tumours of the maxillofacial bones are subdivided in benign and malignant maxillofacial bone and cartilage tumours, fibro-osseous and osteochondromatous lesions as well as giant cell lesions and bone cysts. The histology always needs to be evaluated considering also the clinical and radiological context which remains an important cornerstone in the classification of these lesions. Nevertheless, the diagnosis of maxillofacial bone tumours is often challenging for radiologists as well as pathologists, while an accurate diagnosis is essential for adequate clinical decision-making. The integration of new molecular markers in a multidisciplinary diagnostic approach may not only increase the diagnostic accuracy but potentially also identify new druggable targets for precision medicine. The current review provides an overview of the clinicopathological and molecular findings in maxillofacial bone tumours and discusses the diagnostic value of these genetic aberrations.

## Benign maxillofacial bone tumours

**Fig. 1:**
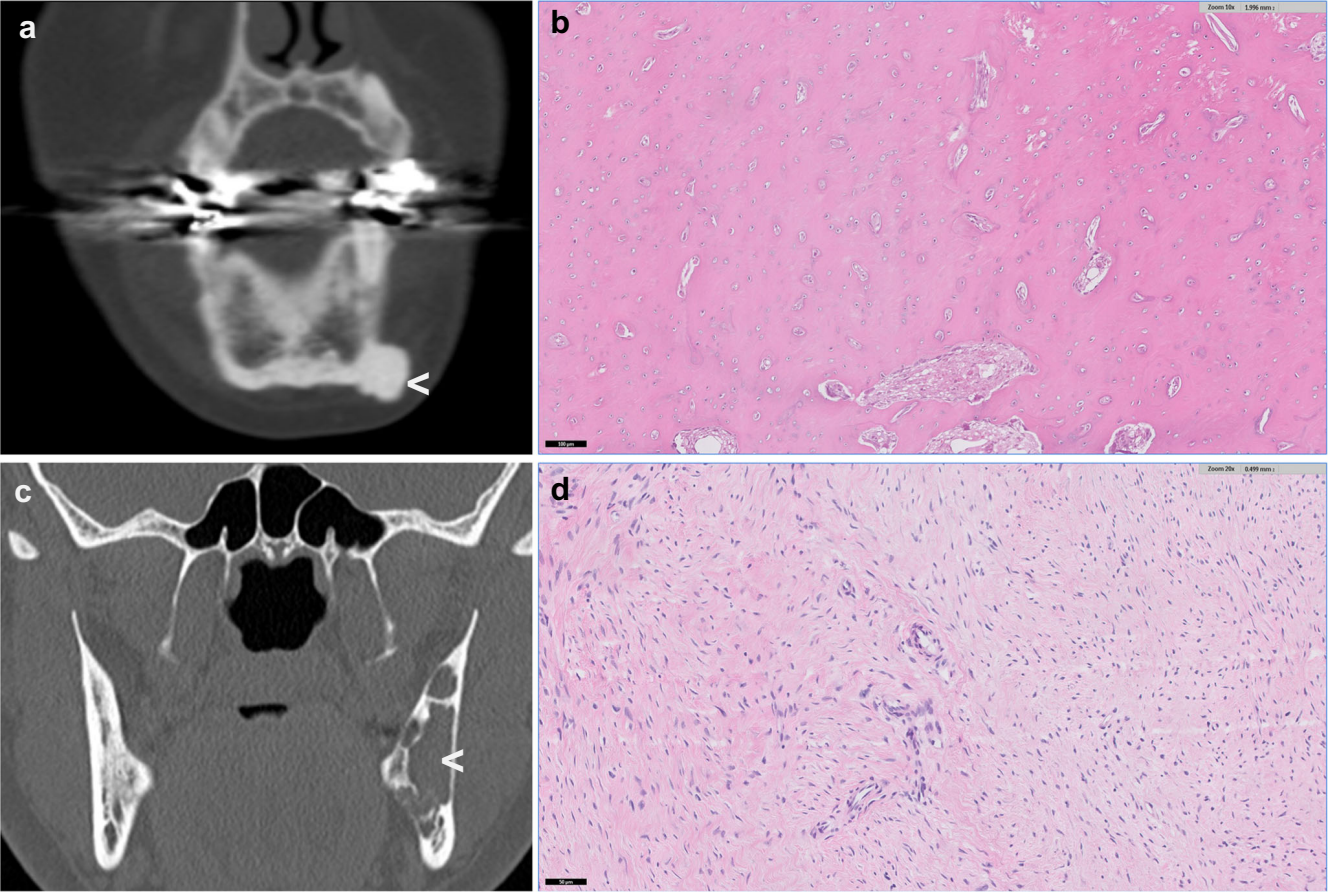
Radiology and morphology examples of benign maxillofacial bone tumours. **a** Coronal reformatted CT image demonstrating the homogenous sclerotic mass of an osteoma arising from the inferior surface of the mandible (arrowhead). **b** HE slide showing osteoma composed of compact mature lamellar bone. **c** Coronal reformatted CT image of a desmoplastic fibroma showing a well-defined osteolytic lesion in the angulus of the mandible on the left side (arrowhead). Cortical erosion and internal septations but no soft-tissue extension. **d** Morphology of a desmoplastic fibroma showing a cellular proliferation of spindle to stellate cells arranged in long fascicles in a collagen-rich matrix

**Fig. 2:**
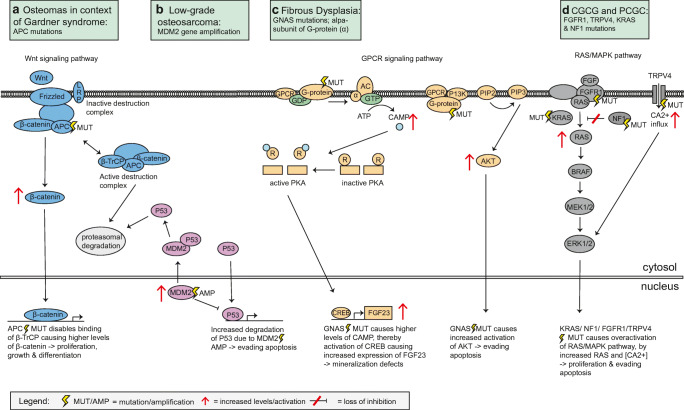
Genetic pathways effected by mutations in maxillofacial bone tumours. **a** Osteomas in context of Gardner syndrome harbour APC mutations. APC mutations disable ubiquitination of β-catenin by β-TrCP, resulting in absence of the active destruction complex and no proteasomal degradation of β-catenin. Consequently, β-catenin levels rise causing cell proliferation, growth and differentiation. **b** MDM2 gene amplification in low-grade osteosarcoma leads to increased proteasomal degradation of P53, as MDM2 enables ubiquitination of P53, resulting in evading apoptosis. **c** The GNAS gene encodes subunit of G protein (α) and is important in the GCPR signalling pathway. GNAS mutation in fibrous dysplasia leads to increased levels of CAMP, thereby activation of CREB causing increased expression FGF23, resulting in mineralisation defect. GNAS mutations can also cause elevated levels of AKT, leading to evading apoptosis. **d** FGFR1, TRPV4 and KRAS gain-of-function mutations lead to an increase activation ERK1/2 and thus overactivation of the RAS/MAP kinase pathway in central giant cell granuloma or peripheral giant cell granuloma. NF1 loss-of-function mutations in context of neurofibromatosis type 1 result in loss of inhibition of RAS

Osteoma is a benign neoplasm composed of mature bone almost exclusively found in the maxillofacial bones and more commonly in the mandible (condyle) than in the maxilla or sino-orbital bones. There is a male predominance, and most osteomas seem to occur in the third to fifth decades of life. Osteomas can be located on the surface of the bone or within the medullary cavity with most central osteomas developing in the mandible or sino-orbital bones (Fig. 1a). Typically, osteomas are composed of compact and mature lamellar bone that merges with the pre-existing bone (Fig. 1b). In the fronto-ethmoid region, osteomas can contain fibrous stroma and osteoblastoma-like areas. With adequate radiological correlation, the diagnosis of an osteoma should not cause great difficulty for a pathologist. In rare cases of osteoma morphologically mimicking osteoblastoma, additional IHC or FISH testing for *FOS* can rule out osteoblastomas that are known to harbour *FOS* rearrangements [1, 2]. The genetic background of sporadic osteomass is unknown, and multiple osteomas can occur in a syndromal context, including Gardner syndrome (a syndrome known to be part of the extraintestinal manifestation of the familial adenomatous polyposis (FAP) spectrum, Fig. 2a) [3]. FAP is a rare genetic disorder with autosomal dominant inheritance caused by mutations in the tumour suppressor gene *APC* [3, 4]. Frameshift and nonsense mutations account for more than 90% of *APC* mutations, and different types of germ line mutations cause different phenotypes. Overall, 65–80% of patients with FAP develop osteomas [3].

**Table 1:** Summary of clinicopathological and molecular findings in maxillofacial bone tumors

		Most frequent age of diagnosis	Gnathic location	Preferred craniofacial sublocation	Radiodensity	Border definition	Tooth displacement/resorption	Cortical destruction	Typical Radiologic Features	Morphological findings	Molecular findings
Benign maxillofacial bone tumours											
*Osteoma*		2nd–5th decade	Mandible > maxilla	Posterior body/Condyle/Paranasal sinus	RO	WD	Possible	No	Homogenously dense, ivory-like sclerotic mass with sharp borders attached to the cortex	Compact mature lamellar bone that merges with the pre-existing bone	*APC mutations (Gardner Syndrome)*
*Desmoplastic fibroma*		1st decade	Mandible > maxilla	Posterior jaw	RL	PD > WD	Possible	Possible	Sunburst appearance possible in case of destruction of the cortex and soft tissue extension	Slender spindle cells arranged in fascicles in a collagen-rich matrix	*Infrequently CTNNB1 and APC mutations*
Malignant maxillofacial bone tumours											
*Chondrosarcoma*		N/A	Maxilla > = mandible	Anterior maxilla/posterior mandible/sino-nasal	Mixed/RL	PD	Possible	Possible	Scattered radiopaque foci, periodontal ligament widening, sunburst appearance	Permeative growth, atypical cartilaginous matrix, pending on the grade cellularity, spindling and atypia of the cells increases	*IDH1/2 mutations*
*Osteosarcoma*		3rd–4th decade	Mandible > = maxilla	N/A	Mixed/RL/RO	PD	Possible	Possible	Sunburst appearance, periodontal ligament widening, cotton wool/orange peel appearance	Permeative growth, highly atypical cells (high grade OS) producing tumour osteoid and more bland spindle cells producing osteoid in ow-grade OS. Predominant matrix of neoplastic bone or cartilage matrix defines osteoblastic osteosarcoma or chondroblastic osteosarcoma subtype	*Complex aberrations due to chromosomal instability, MDM2 amplifications preferentially in low-grade tumours*
Fibro-osseous lesions											
*Ossifying fibroma (OF)*	*Cemento-OF (COF)*	3rd–4th decade	Mandible > Maxilla	Tooth-bearing areas/posterior bod/sino-nasal	RL/Mixed/RO	WD with RL rim	Possible	No	Centrifugal growth pattern maintaining a spherical configuration associated expansion of surrounding cortical bone	Variable mixture of monomorphic fibroblastic spindle cells and immature bone trabeculae as well as cementum-like material	*CDC73 mutations, MEN1 deletions*
	*Juvenile psammomatoid OF (JPOF)*	2nd decade	Maxilla > mandible	Extra-gnathic: periorbital, frontal and ethmoid sinus	RL/Mixed/RO	WD by RO border	Possible	Possible	Ground glass radio-opaque structure with composition varying on degree of calcification	Characteristically small spherical ossicles of bone rimmed with osteoblasts	N/A
	*Juvenile trabecular OF (JTOF)*	2nd decade	Maxilla > mandible	N/A	Mixed>RL	WD by RO border	Possible	Possible	Primarily radiolucent with irregular and scattered calcification	Mineralised tissue appears immature and woven in structure	N/A
*Fibrous dysplasia*		1st–2nd decade	Maxilla > mandible	Gnathic and sphenoid bones most affected; mostly unilateral and monostotic form	RL/mixed/RO	PD	Not common	No	Ground-glass opacification with density related to maturity may involve adjacent bone	Immature woven bone with a curvilinear architecture, usually no osteoblastic rimming, Sharpey’s fibres radiate perpendicularly from the immature matrix	*GNAS mutations*
*Cemento-osseous dysplasia (COD)*	*Periapical COD*	3rd–5th decade	Mandible	Periapical of mandibular incisors	RL/mixed/RO	WD/PD	No	No	Density varies according stage of maturation; border may have sclerotic periphery and radiolucent internal rim	Microscopic findings are identical within the three subgroups of COD showing a fibroblastic stroma with variable cellularity and a heterogeneous osseous component composed of woven bone and cementum-like material	N/A
	*Focal COD*	3rd– 5th decade	Mandible > maxilla	Posterior mandible periapical or edentulous sites	RL/mixed/RO	WD/PD	No	No	Density varies according stage of maturation; border may have sclerotic periphery and radiolucent internal rim		N/A
	*Florid COD*	4th–5th decade	Mandible >maxilla	Multi-quadrant lesions usually more or less symmetrically in alveolar bone	RL/mixed/RO	WD/PD	No	No	Density varies according stage of maturation; border may have sclerotic periphery and radiolucent internal rim		N/A
*Familial gigantiform cementoma*		1st–2nd decade	Maxilla and mandible	Multi-quadrant lesions crossing the midline involving basal and alveolar processes	Mixed/RO	WD with RL rim	Possible	No	Composition varying on degree of maturation becoming predominantly radio-opaque	Monomorphic spindle cells along with immature bone trabeculae and cementum-like material	*ANO5 (in gnathodiaphyseal dysplasia)*
Giant cell lesions and bone cysts											
*Central giant cell granuloma*		2nd decade	Mandible > maxilla	Anterior and posterior mandible/anterior maxilla	RL	WD > PD	Possible	Possible	May vary from small unilocular lesions to large multilocular expanding lesions with soap bubble appearance	CGCG and PGCG are identical in morphology showing a proliferation of mononuclear spindle-shaped and polygonal cells intermixed with osteoclast-like multinucleated giant cells	*TRPV4, KRAS and FGFR1 mutations*
*Peripheral giant cell granuloma*		3rd–7th decade	Mandible > = maxilla	N/A	N/A	N/A	N/A	N/A	No specific radiologic findings		*KRAS and FGFR1 mutations*
*Cherubism*		1st decade	Mandible > maxilla	Posterior mandible	RL	PD > WD	Possible	Possible	Multiquadrant multilocular radiolucent expansile lesions in maxilla and/or mandible	Morphological findings are not specific and overlap with other giant cell-containing lesions	*SH3BP2 mutations*
*Aneurysmal bone cyst*		1st–2nd decade	Mandible > maxilla	Posterior jaw	RL/mixed	WD/PD	Possible	Possible	Various radiological appearance from unilocular to multilocular radiolucency with honeycomb or soap bubble appearance	Blood-filled cystic spaces separated by fibrous septa with bland and plump spindle cells, intermixed with osteoclast-type giant cells and foci of reactive new bone formation	*USP6 rearrangements*
*Simple bone cyst*		2nd decade	Mandible > > maxilla	Posterior mandible	RL	WD	Not common	No	Dome-like projections scalloping between dental roots	A cavity lined by connective tissue with myxoid changes and deposition of cloud-like collagen deposits	N/A

*RL* Radiolucent; *RO* Radiopaque; *Wd* Well-defined; *Pd* Poorly-defined; *>* more; >= slightly more; > > much more; *N/a* not available

Desmoplastic fibroma is a locally aggressive neoplasm most frequently seen in young adults and adolescents. It accounts for only 0.1% of all primary bone tumours and may involve any bone but most frequently occurs in the body and angle of the mandible (Fig. 1c). The tumour is composed of slender spindle to stellate cells arranged in fascicles or whorls in a collagen-rich matrix (Fig. 1d). The morphology can mimic the spindle cell component of low-grade osteosarcoma but generally lacks neoplastic bone formation. Desmoid-type fibromatosis or more mature forms of fibrous dysplasia without abundant bone formation can also be challenging to differentiate. Additional molecular testing is helpful in differentiating desmoplastic fibroma from low-grade osteosarcoma (MDM2 amplification) and fibrous dysplasia (GNAS mutation). Low-grade fibrosarcomas/myofibroblastic sarcoma shows spindle cells with atypia, anisopleomorphism or mitotic figures that are usually not present in desmoplastic fibroma.

Whether desmoplastic fibroma of bone really represents the intra-osseous counterpart of desmoid-type fibromatosis of soft tissues is still a matter of debate [5]. Limited data is available on the mutation status of *CTNNB1*specifically in desmoplastic fibroma of gnatic location, in one of two desmoplastic fibromas in the mandible, and a *CTNNB1* mutation was detected [6, 7, 8]. Immunohistochemistry to detect nuclear beta-catenin expression is considered to be of limited diagnostic value since staining can be observed in various fibro-osseous maxillofacial lesions (25–40%) except for fibrous dysplasia [9].

## Malignant maxillofacial bone and cartilage tumours

**Fig. 3:**
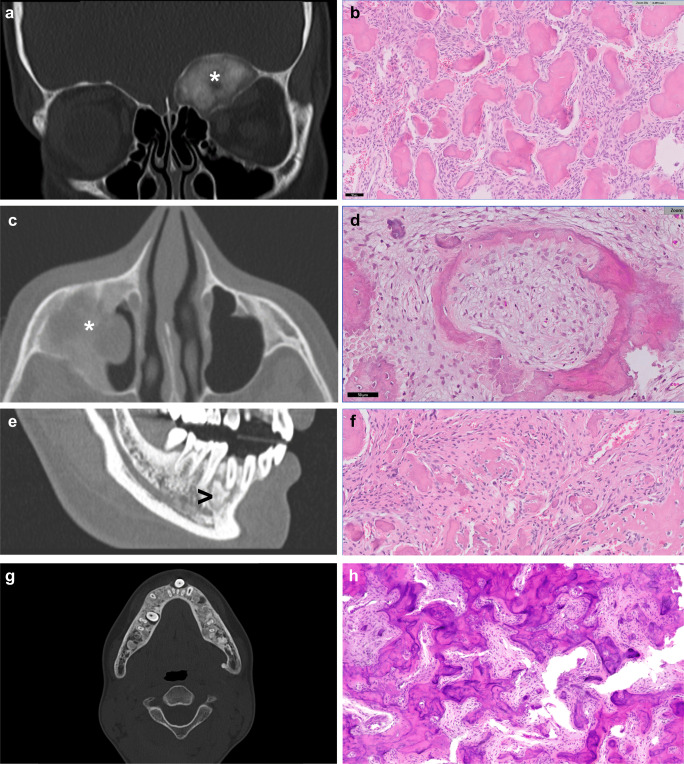
Radiology and morphology examples of malignant maxillofacial bone and cartilage tumours. **a** Coronal T1-weighted MR image with fat suppression after intravenous contrast administration. Typical septonodular enhancement of a chondrosarcoma in the left nasal cavity and maxillary sinus (asterisk). Rim enhancement of the orbital extension (arrowhead). **b** Entrapment of pre-existent bone by neoplastic chondroid matrix with atypical chondrocytes. **c** Axial CT image showing osteolytic mesenchymal chondrosarcoma in the anterior mandible (asterisk). Punctate calcifications within the lesion (arrowhead). Cortical destruction and soft-tissue extension, predominantly on the anterior side (double arrowhead). **d** Typical morphology of a mesenchymal chondrosarcoma with islands of immature-appearing cartilaginous matrix (double arrowhead) and round tumour cells with in the background hemangiopericytoma-like vessels. **e** Curved planar reconstructed CT image of an osteosarcoma demonstrating an ill-defined lesion in the mandible consisting of osteolysis (double arrowhead) and sclerosis caused by tumour mineralisation in the centre (arrowhead). **f** Morphology of chondroblastic osteosarcoma with atypical cellular chondroid matrix (double arrowhead) surrounded by a cellular spindled component producing abundant eosinophilic matrix with focal areas of osteoid (arrowhead) bone matrix

Chondrosarcoma is a malignant bone tumour that produces a cartilaginous matrix. In the jaw bones, chondrosarcomas are exceedingly rare which might be influenced by their development mainly through intramembranous ossification. A larger meta-analysis reported maxillofacial chondrosarcomas to account for 3–4% of all chondrosarcomas although it is impossible to exclude chondroblastic osteosarcomas in all the studies considered that can closely mimic chondrosarcoma [10]. The maxilla and nasal septum are more frequently involved than the mandible (Fig. 3a), and patients of any age can be affected. In most cases, chondrosarcoma shows entrapment of pre-existing bone and/or cortical permeation. Well-differentiated chondrosarcomas (atypical cartilaginous tumour/chondrosarcoma grade 1) show hyaline cartilage with oval to polygonal chondrocytes and frequent binucleation (Fig. 3b). With increasing grade (2 + 3), cellularity, spindling and atypia of the cells increase, and mitotic figures are usually observed.

Chondroblastic osteosarcoma is far more common than chondrosarcoma in the jawbones, and the cartilaginous component in both tumours may morphologically overlap. The diagnosis of gnathic chondrosarcoma thus requires a thorough search to exclude tumour osteoid deposition which is pathognomonic for osteosarcoma. Since 49–61% of chondrosarcomas harbour an *IDH1/2* mutation, additional mutation testing can be helpful to distinguish between these differential diagnoses in which the finding of an *IDH1/2* mutation is diagnostic for chondrosarcoma and excludes chondroblastic osteosarcoma [11–13]. Since not all chondrosarcomas harbour *IDH1/2* mutations, the finding of wild-type *IDH1/2* does not exclude chondrosarcoma. Isocitrate dehydrogenase (IDH) is an enzyme that catalyses the oxidative decarboxylation of isocitrate into alpha-ketoglutarate in the Krebs TCA cycle. Mutations are exclusively found on the arginine residues R132 in *IDH1* and R140 and R172 in *IDH2*. The mutant enzyme acquires the ability to convert α-ketoglutarate into the oncometabolite D-2-hydroxyglutarate (D-2-HG) [14, 15].

Mesenchymal chondrosarcomas are rare, develop in the second to fourth decade of life and are most commonly affecting the craniofacial bones, particularly the jaws (Fig. 3c). The morphology of mesenchymal chondrosarcoma is typically a combination of small blue round cells with hemangiopericytoma-like vessels admixed with islands of immature-appearing cartilaginous matrix (Fig. 3d). Mesenchymal chondrosarcomas generally harbour a *HEY1-NCOA2* or *IRF2BP2-CDX1* gene fusion that can be helpful to distinguish rhabdomyosarcoma or small cell osteosarcoma, particularly in core needle biopsies that lack the chondromatous component [16, 17].

Osteosarcoma is the most common primary malignant tumour of bone most commonly occurring in the metaphyses of long bones in children and adolescents. The fourth most common site of osteosarcoma is the jaw bones (Fig. 3e). Compared to extra-gnatic osteosarcomas, osteosarcomas of the jaw tend to occur 10–20 years later and are preferentially located in the mandible compared to maxillary location [18].

Conventional osteosarcoma is a high-grade intra-osseous tumour defined by highly atypical cells producing tumour osteoid. The morphology of conventional osteosarcoma shows a broad spectrum which is subclassified into osteoblastic (polygonal to epithelioid cells), chondroblastic (highly atypical chondrocyte-like cells) and fibroblastic (highly atypical spindle cells) subtypes depending on the predominating matrix formed (Fig. 3f) in which, for example, a predominant matrix of neoplastic bone (abundant coarse lace-like) or cartilage matrix defines osteoblastic osteosarcoma or chondroblastic osteosarcoma, respectively. The amount of bone matrix varies and can appear lace-like or in the form of irregular mineralised woven bone. The chondroblastic variant is a subtype frequently seen in the jaws and can mimic chondrosarcoma, which is far less common in this location. The finding of convincing tumour osteoid distinguishes chondroblastic osteosarcoma from chondrosarcoma as mentioned previously; additional testing for *IDH1/2* may be helpful to differentiate. Small cell and telangiectatic osteosarcoma are exceptionally rare in the jaws. Low-grade osteosarcoma shows less pleomorphism and more subtle atypical fibroblastic cells with scarce mitotic activity and irregular trabeculae of woven bone. The fibroblastic component may dominate with low to moderate cellularity which can appear similar like fibrous dysplasia. Juxtacortical osteosarcomas of the jaws are exceedingly rare and are histologically identical to their counterparts in the peripheral skeleton.

*MDM2* gene amplification has been reported in more than 60% of low-grade osteosarcomas of the jaw compared to 12% in conventional osteosarcomas (Fig. 2b) [19, 20], whereas immunohistochemistry against MDM2 (and CDK4) is considered sensitive but not specific, and only MDM2 amplification detected by FISH strongly argues in favour of osteosarcoma and can be helpful to distinguish low-grade osteosarcoma from its fibro-osseous mimics. In contrast to low-grade osteosarcoma, high-grade osteosarcomas have complex karyotypes with abundant structural and numerical aberrations frequently resulting from chromothripsis [21].

Distinguishing low-grade osteosarcoma from benign fibro-osseous lesions of the jaw including ossifying fibroma, fibrous dysplasia and cemento-osseous dysplasia may be challenging when imaging is not complete and biopsy material is scarce. Entrapment of pre-existent bone is never observed in benign fibro-osseous lesions and therefore a distinguishing morphological feature. Also invasive growth into cortical bone is a feature of osteosarcoma and absent in benign lesions. Nevertheless, the rather bland cytonuclear appearance of low-grade osteosarcoma can be a pitfall, and the clinical and radiologic context have always to be considered before making a diagnosis.

## Fibro-osseous lesions

Fibro-osseous lesions of the jaw include ossifying fibroma, fibrous dysplasia and cemento-osseous dysplasia that can show overlapping histological features but are usually easy to differentiate if morphology, imaging and clinical presentation are considered together. An interdisciplinary approach is therefore mandatory to accurately classify these lesions.

**Fig. 4:**
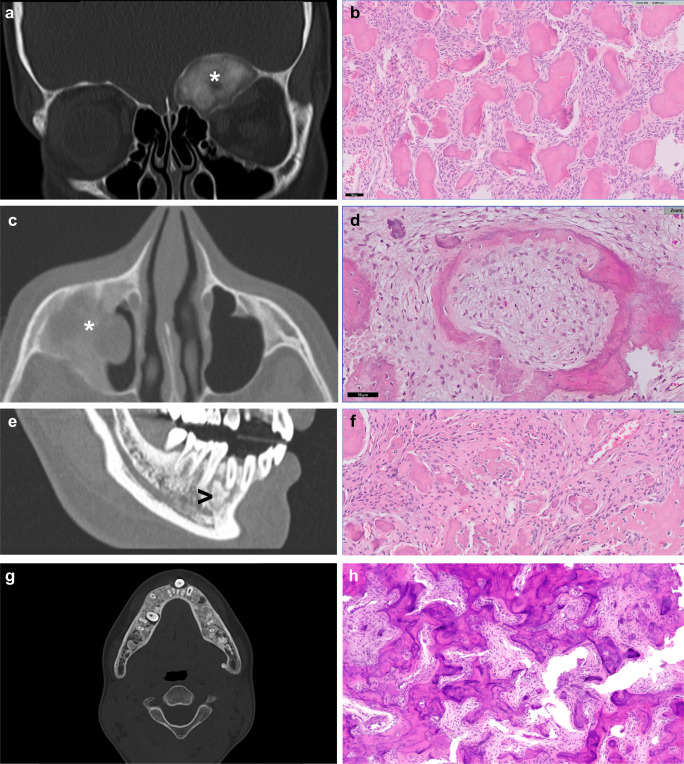
Radiology and morphology examples of fibro-osseous lesions. **a** Coronal reformatted CT image of a patient with a juvenile psammomatoid ossifying fibroma showing a well-defined ossifying lesion arising from the superior margin of the left orbit (asterisk). The lesion is surrounded by a thin ossified shell. **b** Morphology of juvenile psammomatoid ossifying fibroma showing a cellular bland spindle cell component and characteristically small spherical ossicles (psammomatoid bodies) of bone rimmed with more flattened osteoblasts. **c** Axial CT image of fibrous dysplasia located in the right maxillary sinus showing a mass (asterisk) with typical ground-glass appearance without cortical interruption or soft-tissue extension. **d** Typical morphology of fibrous dysplasia with woven bone trabeculae mimicking Chinese script letters and Sharpey’s fibres that radiate into the surrounding cellular stroma. **e** Reformatted CT image of a (focal) cemento-osseous dysplasia showing the ossifying lesion near the root of right first premolar in the mandible (arrowhead). **f** Morphology of a cemento-osseous dysplasia showing a fibroblastic stroma with variable cellularity and a heterogeneous osseous component composed of woven bone and more cementum-like material. **g** CT scan of a patient with familial gigantiform cementoma showing bilateral massive involvement. **h** Familial gigantiform cementoma showing irregular trabeculae of woven bone and monomorphic spindle cells

Ossifying fibroma is a benign neoplasm affecting the jaws and craniofacial bones and comprises three subtypes: conventional cemento-ossifying fibroma (COF), juvenile trabecular ossifying fibroma (JTOF) and juvenile psammomatoid ossifying fibroma (JPOF). COF is considered a benign odontogenic tumour and exclusively develops in the tooth-bearing parts of the jaws. It has a preference to occur in the mandible, favouring molar and pre-molar regions in the third to fourth decade of life with a female predilection. JTOF and JPOF most commonly occur in the second decade without a gender predilection but can occur also later in life. Both subtypes can develop in extra-gnathic bones (Fig. 4a), whereas JPOF is most common in the sinuses, particularly the ethmoid sinus, and JTOF general affects the jaws with the maxilla representing the most prevalent site. JTOF and JPOF can show more rapid growth and expansion causing facial disfigurement, visual changes and sinus dysfunction. In contrast, COF usually presents as a well-defined and slowly progressing mass that can nevertheless reach considerable sizes if left untreated.

Morphologically, COF is composed of a variable mixture of monomorphic fibroblastic spindle cells and immature bone trabeculae as well as cementum-like material. Osteoblastic rimming is a prominent feature in COF, and the stroma may vary in cellularity; significant atypia, however, does not occur. Typically, COF is surrounded by a thin layer of connective tissue preventing lesional and pre-existing bone to fuse. In JTOF, the stroma can be dense as well, might contain mitotic figures and infiltrates the surrounding bones. The mineralised tissue seems to develop directly from the stromal cells and appears immature and woven in structure. Usually, it is not rimmed by prominent osteoblasts, and occasionally, groups of osteoclastic giant cells can be observed. JPOF characteristically shows small spherical ossicles of bone rimmed with more flattened osteoblasts (Fig. 4b). These small ossicles also referred to as psammomatoid bodies and may coalesce to form larger matrix formations.

The molecular pathogenesis of ossifying fibroma and its subtypes is unclear. Some studies detected mutations in *CDC73* (*HRPT2*) in patients with hyperparathyroidism-jaw tumour (HPT-JT) syndrome. HPT-JT is an autosomal dominant disorder in which 25–50% of patients develop COF and practically the only cause of multifocal COF [22]. *CDC73* is a tumour suppressor gene encoding the parafibromin protein, a transcriptional and post-transcriptional regulator targeting both the Wnt/beta-catenin and Hedgehog pathways. However, in the pathogenesis of sporadic COF, *CDC73* mutations seem to play only a minor role as the reported frequency was only 5% [2]. In a recently described mouse model, deletion of the *MEN1* gene resulted in COF development, further underlining the potential role of hyperparathyroidism or at least endocrine abnormalities in the pathogenesis of COF [23]. A study performed by Pereira et al. in 2018 showed activation of the Wnt/beta-catenin pathway in cemento-ossifying fibromas without evidence of oncogenic mutations in the *CTNNB1* or *APC* genes. Further studies are needed to clarify the precise involvement of this signalling cascade in COF [24]. Furthermore, dysregulated microRNAs targeting *EZH2, XIAP, MET* and *TGFBR1* in combination with the expression of Notch signalling molecules have been described in COF [25, 26]. In any case, the molecular studies performed so far did not identify a recurrent genetic aberration in human COF that could be used as a diagnostic marker. Likewise, molecular studies in JTOF and JPOF are scarce, and rearrangements involving 2q and Xq (t(X;2)(q26;q33)) have been described in few selected cases of JPOF [27, 28].

Fibrous dysplasia (FD) can affect any bone but frequently involves the maxillofacial skeleton. It can develop as monostotic or polyostotic disease, in the McCune-Albright syndrome also together with skin lesions and endocrine disorders. Notably, FD affecting adjacent craniofacial bones is still considered as monostotic disease. In the jaws, FD occurs more often in the maxilla (Fig. 4c) than in the mandible and is primarily a disease of growing bones identified in children and adolescents (although it can manifest later in life).

The morphology of FD consists of a mature fibrous tissue with bland-appearing fibroblastic cells and immature woven bone formation, often with a peculiar curvilinear architecture. There is usually no osteoblastic rimming. In connective tissue stains (e.g. van Gieson), Sharpey’s fibres radiate perpendicularly from the immature matrix into the surrounding stroma (Fig. 4d). Over time, the lesional bone can undergo maturation to lamellar bone. Remarkably, the lesional matrix in FD typically fuses with the adjacent normal bone which is a distinguishing feature to COF.

FD is an example of a fibro-osseous lesion in which the underlying molecular pathogenesis has been identified, namely, a postzygotic-activating mutation in the *GNAS* gene encoding the alpha-subunit of the stimulatory G protein Gs (Fig. 2c) [29, 30]. *GNAS* mutations, depending on the molecular detection method used, can be identified in 45%–88% of FD cases and are not present in other fibro-osseous lesions such as cemento-ossifying fibroma, cemento-osseous dysplasia or low-grade osteosarcoma [29, 30].

Cemento-osseous dysplasia (COD) is the most common benign fibro-osseous lesion of the jaw occurring exclusively in the tooth-bearing areas, more often in the mandible and predominantly in middle-aged (black) women (Fig. 4e). COD may be under-represented in surgical pathology because the diagnosis can be made already clinically and radiologically and does not require bioptic confirmation or specific treatment. Three subtypes can be distinguished: (1) periapical cemento-osseous dysplasia (localized in the periapical regions of the mandibular incisors), (2) focal cemento-osseous dysplasia (monofocally, anywhere in the jaws except for the mandibular incisors) and (3) florid cemento-osseous dysplasia (multifocally, often multi-quadrant involvement). Biopsy is by some experts even contraindicated obsolete since it increased the risk of secondary osteomyelitis which can be difficult to treat in the COD context.

Although COD is subdivided based on the pattern of involvement, the microscopic findings are identical within the three subgroups, and thus subdivision is based on clinical and radiological features. COD is not encapsulated and shows a fibroblastic stroma with variable cellularity and a heterogeneous osseous component composed of woven bone and cementum-like material (Fig. 4f). The stroma is well vascularised and may harbour extravasated red blood cells. Osteoblastic rimming is uncommon, and as lesions mature, the woven bone and cementum coalesce to larger formations of heavily mineralised matrix. COD is usually self-limiting, and expansion of bone is uncommon but can occur in the florid subtype. The lesion is unequivocally benign, and malignant transformation does not occur.

So far, molecular data on cemento-osseous dysplasia has not been published. Although COD is considered a benign and presumably non-neoplastic lesion, the recent finding of genetic driver events in giant cell granuloma might suggest a genetic aberration to underlie also COD, but this assumption remains purely hypothetical.

Familial gigantiform cementoma is a very rare fibro-osseous lesion of the maxillofacial bones characterized by early onset of multifocal/multiquadrant progressively expansive lesions (Fig. 4g). The morphology overlaps with conventional cemento-ossifying fibroma and consists of monomorphic spindle cells along with immature bone trabeculae and cementum-like material (Fig. 4h). Familial gigantiform cementoma can be inherited in an autosomal dominant fashion but does not necessarily present with a familial background. Gigantiform cementoma is also described in rare generalized skeleton syndromes like gnathodiaphyseal dysplasia. In the context of gnathodiaphyseal dysplasia, mutations in the anoctomin 5 (*ANO5*) gene have been detected in gigantiform cementoma [31]. Whether mutations in *ANO5* also play a role in patients with familial gigantiform cementoma without a generalized skeleton syndrome remains to be elucidated.

## Giant cell lesions and bone cysts

Central giant cell granuloma (CGCG), peripheral giant cell granuloma, cherubism and aneurysmal bone cyst are examples of mesenchymal tumours in the jaws that contain giant cells and show overlapping clinical and morphological features.

**Fig. 5:**
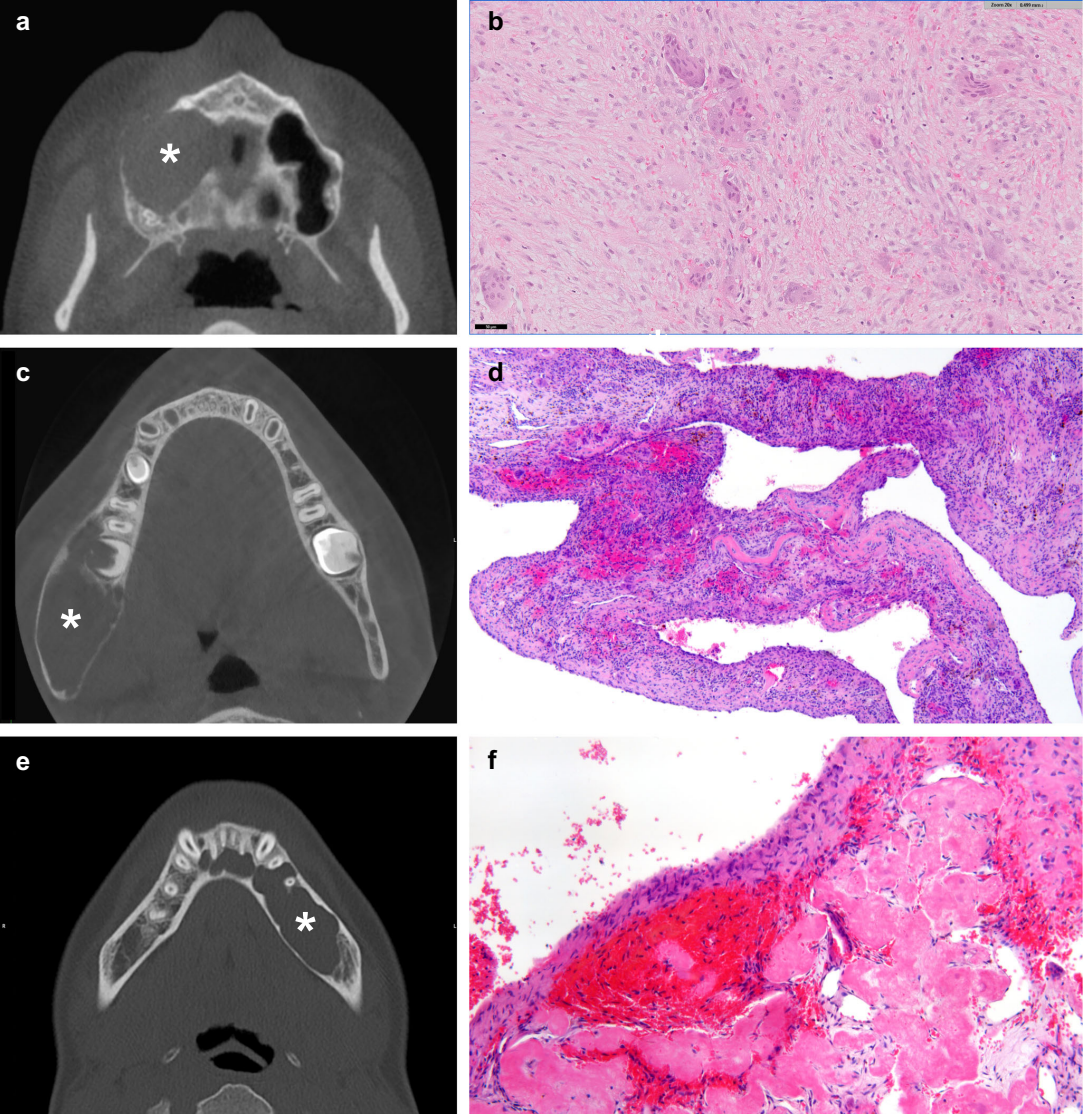
Radiology and morphology examples of giant cell lesions and bone cysts. **a** Central giant cell granuloma axial CT image with an expansile unilocular osteolytic lesion (asterisk) arising from the right maxilla surrounded by a thin bony shell and extending into the right maxillary sinus. **b** Morphology of giant cell showing a proliferation of mononuclear spindle-shaped and polygonal cells intermixed with osteoclast-like multinucleated giant cells and haemorrhage. **c** CT image showing an aneurysmal bone cyst in the right mandible (asterisk) with well-delineated locular radiolucencies. **d** HE stain shows cystic spaces separated by fibrous septa with proliferations of fibroblastic type spindle cells intermixed with osteoclast type of giant cells and foci of osteoid. **e** CT showing a simple bone cyst in the left mandible (asterisk) with well-defined radiolucency extending between the roots of associated teeth without root resorption. **f** The histology of simple bone cysts shows a cavity filled with blood lined by connective tissue with deposition of collagen

Central giant cell granuloma (CGCG) is a benign localized, sometimes aggressive osteolytic lesion of the jaws accounting for 10% of benign gnathic tumours. CGCG most often occurs in females aged < 20 years. The lesions are more frequently located in the anterior part of the mandible (Fig. 5a). Multiple lesions have been described in Noonan syndrome, LEOPARD syndrome, neurofibromatosis type 1 (NF1) and rarer forms of RASopathies [32]. The typical morphology is characterized by an unencapsulated proliferation of mononuclear spindle-shaped and polygonal cells intermixed with osteoclast-like multinucleated giant cells. There is a vascular background with haemorrhage and haemosiderin pigment deposits (Fig. 5b). The morphology overlaps with other giant cell-containing lesions such as giant cell tumour of bone, solid variant of aneurysmal bone cyst, cherubism and “brown” tumours occurring in the context of hyperparathyroidism. CGCG lacks the characteristic *H3F3A* mutation which is identified in > 95% of giant cell tumours of bone [33–35]. This underlines the idea that although giant cell tumour of bone may have morphological similarities with CGCG, it clearly represents a distinct entity that practically does not exist in the jawbones. Instead, *TRP4, KRAS* and *FGFR1* gain-of-function mutations have recently been identified in CGCG suggesting a pathogenetic relationship to non-ossifying fibroma (NOF) of bone [36–38]. Notably, in patients with Jaffe-Campanacci syndrome and oculoectodermal syndrome, patients can develop both CGCG and NOF which therefore belong to the phenotypic spectrum of specific RASopathies (Fig. 2d) [36]. *TRPV4* is involved in promoting differentiation, modulating vascular function and inhibiting osteoclast apoptosis [39]. This gain-of-function mutation effect is reflected in the classical morphology of CGCG with mononuclear cells intermixed with many osteoclast-type giant cells in a haemorrhagic vascular background. *KRAS* mutations in CGCG most frequently involve codon 12 which is the most frequently mutated exon of *KRAS* in neoplasia in general [40]. In 8% of cases analysed, *KRAS* and *TRPV4* were both mutated, in contrast with *FGFR1* mutations that were mutually exclusive with *KRAS* and *TRPV4* mutations [36]. *FGFR1* is involved in bone growth and remodelling, and mutations in this gene have also been described in several types of cancer [41]. Histologic findings together with the clinical and radiological context are usually sufficient to classify CGCG, but occasionally testing for *TRP4, KRAS or FGFR1* mutations might be helpful in making a final diagnosis, particularly in excluding solid variants of aneurysmal bone cyst without *USP6* rearrangement and “brown” tumours. Importantly, patients with CGCG might benefit from MEK inhibitors in recurrent settings or patient with large tumours requiring mutilating surgery.

Opposed to intra-osseous CGCG, peripheral giant cell granuloma (PGCG, giant cell epulis) is the most frequent giant cell-containing lesion of the oral soft tissue. PGCG more commonly affects the gingiva or edentulous alveolar ridge in the mandible but may involve the maxilla as well. PGCG have historically considered to be reactive lesions as a result of chronic irritation and share an identical morphology with CGCG. Recently, however, activating mutations in the MAP-kinase signalling pathway have been described in a series of 21 PCGC as well, including *KRAS* (71%) and *FGFR1* (10%) mutations [36]. These results indicate that PGCG and CGCG are different manifestations of the same biological disease.

Cherubism is an autosomal dominantly inherited disease than can typically affect multiple quadrants of the jaws, often more pronounced in the mandible compared to the maxilla. The morphological findings are not specific and overlap with other giant cell-containing lesions in the jaw such as CGCG. The typical clinical stigmata with facial disfigurement due to expansile jaw lesions potentially leading to the apparent upward gaze of the eyes, resembling Cherub angels in Renaissance paintings, together with the detection of mutations in the *SH3BP2* gene, found in almost 80% of cases, allows the diagnosis of cherubism [42]. The gene *SH3BP2* (SH3 domain-binding protein 1) is located on chromosome 4p16.3 and encodes for an adaptor protein involved in osteoclast differentiation and bone remodelling [42]. The reasons for the exclusive predilection for the jaws, the absence of systemic disorders and the frequent regression after puberty in most cherubism cases are still unresolved.

Aneurysmal bone cyst (ABC), especially the solid variant, harbours overlapping morphological features with the previously discussed giant cell-containing entities in the jawbones. ABC is a locally aggressive, expansile and benign neoplasm of bone composed of multiloculated blood-filled cystic spaces. It is rather rare in the craniofacial bones, and only 1.5% of all cases occur in the jaws (Fig. 5c). More than 60% of ABCs in the maxillofacial bones are located in the mandible, frequently in the posterior region, and the majority of cases occur in individuals younger than 30 years of age. The blood-filled cystic spaces of ABC are separated by fibrous septa which are composed of cellular proliferations of bland- and plump-appearing fibroblastic spindle cells, intermixed with osteoclast type of giant cells and foci of reactive new bone formation (Fig. 5d). Characteristically, the fibrous septa are lined by more flattened lesional cells, and the bone often appears basophilic (so-called blue bone). ABC contains cytogenetic rearrangements of the *USP6* (ubiquitin specific peptidase 6/Tre-2) gene localized on chromosome 17p13. The most common translocation described (30%), including for maxillofacial lesions, leads to fusion of the *USP6* with *CDH11* (cadherin 11) genes which results in upregulation of *USP6* [43–47]. Other reported fusion genes in ABC of non-gnatic location include *PAFAH1B1*, *RUNX2*, *TRAP150, ZNF9, OMd* and *COL1A1* [47]. The finding of *USP6* rearrangements in a giant cell-containing bone lesion of the jaw confirms the diagnosis of primary ABC, ruling out other giant cell-containing bone lesions and secondary ABC. For example, ABC-like areas can be observed in other bone tumours of the jaw including fibrous dysplasia, ossifying fibroma or CGCG and are referred to as secondary ABC. Additionally, solid variant of ABC exists which is impossible to distinguish from CGCG or so-called brown tumours caused by hyperparathyroidism. Differentiating primary from secondary ABC sometimes requires additional molecular testing.

Simple bone cysts are decisively more common in gnatic bones than ABC although they more frequently occur in the metaphysis of long bones in children and adolescents. If located in the gnatic bones, they show a predilection for the mandibular body [48]. Generally, simple bone cysts are asymptomatic and are an incidental findings during routine examination. Radiologically, they present as well-defined radiolucencies frequently extending between the roots of associated teeth without resorption or displacement (Fig. 5e). The histology of simple bone cysts shows a cavity lined by connective tissue with myxoid changes and deposition of cloud-like collagen deposits (Fig. 5f) [49]. No molecular studies have been performed in simple bone cysts so far.

## Conclusions

Since molecular data are scarce in most maxillofacial bone tumours, diagnostic decision-making still heavily relies on the correlation between radiology and morphology. Since clinical and morphological aspects may overlap, especially between the fibro-osseous lesions of the jaw, a definitive diagnosis generally requires clinical and radiological correlation.

Evidently, *IDH1/2* and *GNAS* mutations are examples of useful molecular markers pathognomonic for chondrosarcoma and fibrous dysplasia, respectively. Likewise *MDM2* amplifications for the diagnosis of low-grade osteosarcoma, *HEY1* fusions in case of mesenchymal chondrosarcoma and *USP6* fusions in aneurysmal bone cyst. Central or peripheral giant cell granulomas belong to a broad spectrum of diseases with dysregulation of the MAP-kinase pathway which may have more impact on patient treatment with the use of MEK inhibitors in the future. For other entities including cemento-osseous dysplasia or juvenile variants of ossifying fibroma, the molecular composition is unknown. Hopefully in the near future by using next-generation sequencing platforms, driver events will be identified and translated into diagnostic, prognostic or therapeutic markers, eventually making patient with maxillofacial bone tumours more eligible for targeted therapy.
